# The Simultaneous Occurrence of Acute ST-Elevation Myocardial Infarction, Acute Ischemic Stroke, and Pulmonary Embolism

**DOI:** 10.7759/cureus.44222

**Published:** 2023-08-27

**Authors:** Homayoon Lodeen, Saliman Esmati, Tetyana Okan, Azeem Arastu, Dora Vilendecic, Gagandeep Singh, Aditya Mangla, Pouria Moshayedi, Zoran Lasic

**Affiliations:** 1 Internal Medicine, Jamaica Hospital Medical Center, New York, USA; 2 Internal Medicine, Jamaica Hospital Medical Center, New York , USA; 3 Cardiology, Northwell Health Lenox Hill Hospital, New York, USA; 4 Interventional Neurology, Jamaica Hospital Medical Center, New York, USA

**Keywords:** rare case report, simultaneous occurrence, stroke, myocardial infarction, pulmonary embolism (pe), acute ischemic stroke (ais), st-elevation myocardial infarction (stemi)

## Abstract

Acute ST-elevation myocardial infarction (STEMI), acute ischemic stroke (AIS), and acute pulmonary embolism (PE) are life-threatening conditions, each posing a high risk of morbidity and mortality. When all three of these acute conditions occur simultaneously, the overall prognosis for the patient becomes considerably worse. We report a case of a 70-year-old woman who presented to the emergency department (ED) with a triad of acute STEMI, AIS, and PE as a consequence of atherosclerotic heart disease, atrial fibrillation, and a prolonged transatlantic flight. The diagnoses were promptly confirmed through emergent coronary and cerebral angiography, along with a computerized tomography pulmonary angiogram (CTPA). The patient underwent a combination of medical therapy and endovascular thrombectomy. However, she later developed a subarachnoid hemorrhage and eventually progressed to brain death.

## Introduction

The simultaneous manifestation of acute myocardial infarction (AMI) and acute ischemic stroke (AIS) is notably rare, with an incidence rate of just 0.009% according to research by Yeo et al. [1]. The addition of an acute pulmonary embolism to this already unusual combination makes the occurrence extraordinarily rare. As documented in medical literature, the infrequent trio of AMI, AIS, and acute pulmonary embolism often has a common origin in systemic disease complications or a single source of embolism [2]. What sets this case apart is the simultaneous origination of emboli from both venous and arterial sources. There was no indication that the mechanism of paradoxical embolism was a link for the single source from either the venous or arterial system. Moreover, on the arterial side, AIS and STEMI appear to have two different mechanisms: embolism for the AIS and atherosclerotic cause for STEMI.

## Case presentation

**Figure 1:**
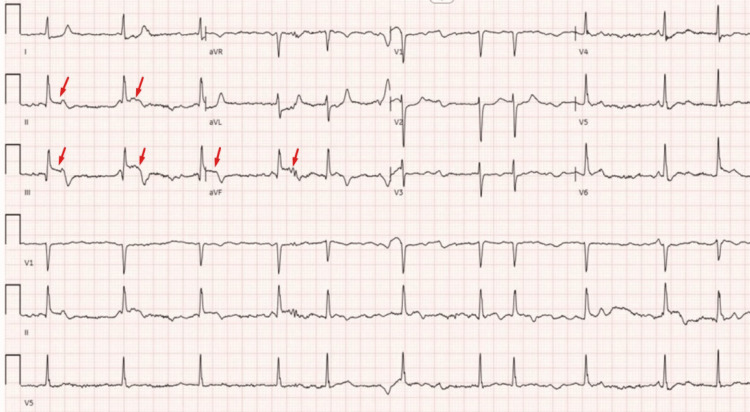
Twelve-lead electrocardiogram at presentation showing atrial fibrillation and acute inferior ST-elevation myocardial infarction.

A 70-year-old woman, with no prior known medical history, was transported to the emergency department (ED) by emergency medical services after presenting with chest pain and an EKG that indicated an acute inferior wall STEMI. The patient had been on a 13-hour transatlantic flight and did not show any symptoms when she initially boarded the plane. She complained of substernal chest pain two hours before landing and suffered two seizure episodes in the ambulance while being transported to the hospital. The patient arrived at the ED in an unresponsive state, exhibiting agonal respiration. She was promptly intubated for immediate respiratory support. The patient's blood pressure could not be recorded; thus, a treatment regimen involving norepinephrine and dopamine was promptly started with an improvement of blood pressure to 100/60 mmHg. EKG showed atrial fibrillation with acute inferior wall ST elevation (Figure 1), and the level of troponin was elevated to 0.38 ng/mL (normal range <0.033 ng/mL), trending up to 62.500 ng/mL within the next nine hours.

**Figure 2:**
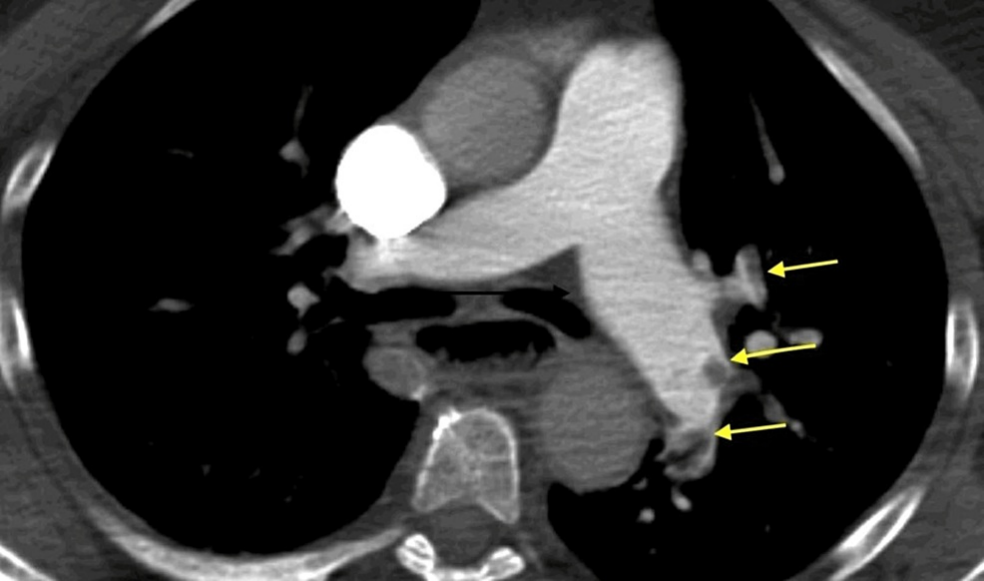
CT pulmonary angiogram with yellow arrows pointing at pulmonary emboli in the left pulmonary artery. CT, computed tomography

**Figure 3:**
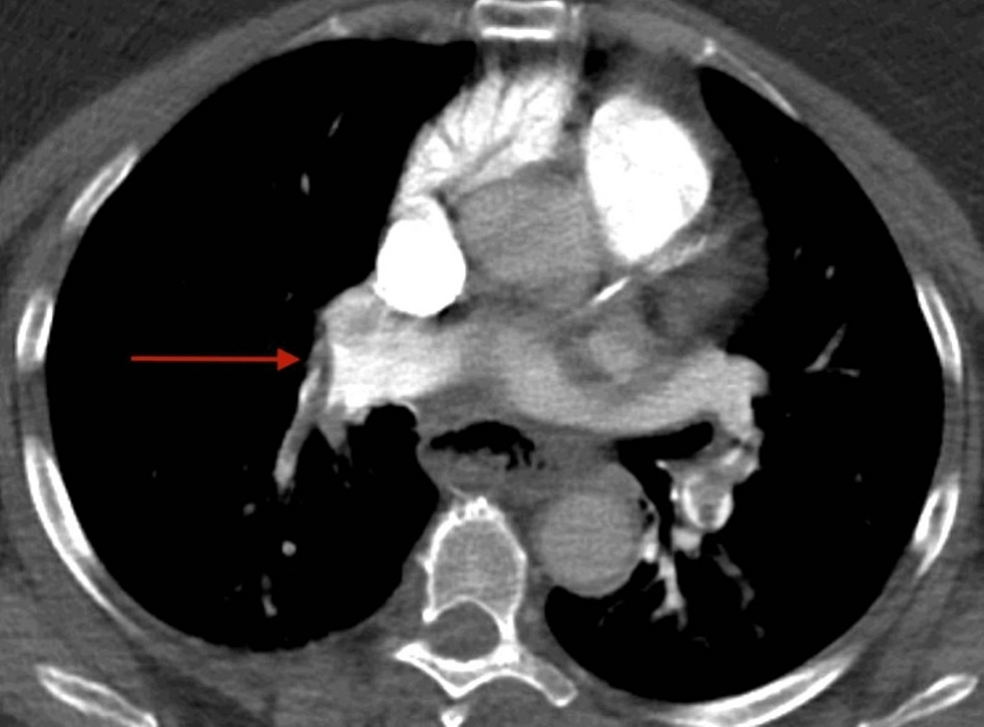
CT pulmonary angiogram showing a red arrow pointing at a pulmonary embolus in the right pulmonary artery. CT, computed tomography

D-dimer was elevated to 25,310 (normal range, 119-500). The patient received heparin 5,000 units, aspirin 325 mg, and ticagrelor 180 mg before a planned primary percutaneous coronary intervention (PCI). Because of a significant shift in her mental state and respiratory distress, the patient underwent a noncontrast computed tomography (CT) scan of the head on her way to the catheterization laboratory. The scan revealed acute bilateral occlusions of the middle cerebral arteries. Additionally, a CT angiogram of her pulmonary arteries was performed, which unveiled acute, bilateral pulmonary emboli in the lower lobes (Figures 2-3).

**Figure 4:**
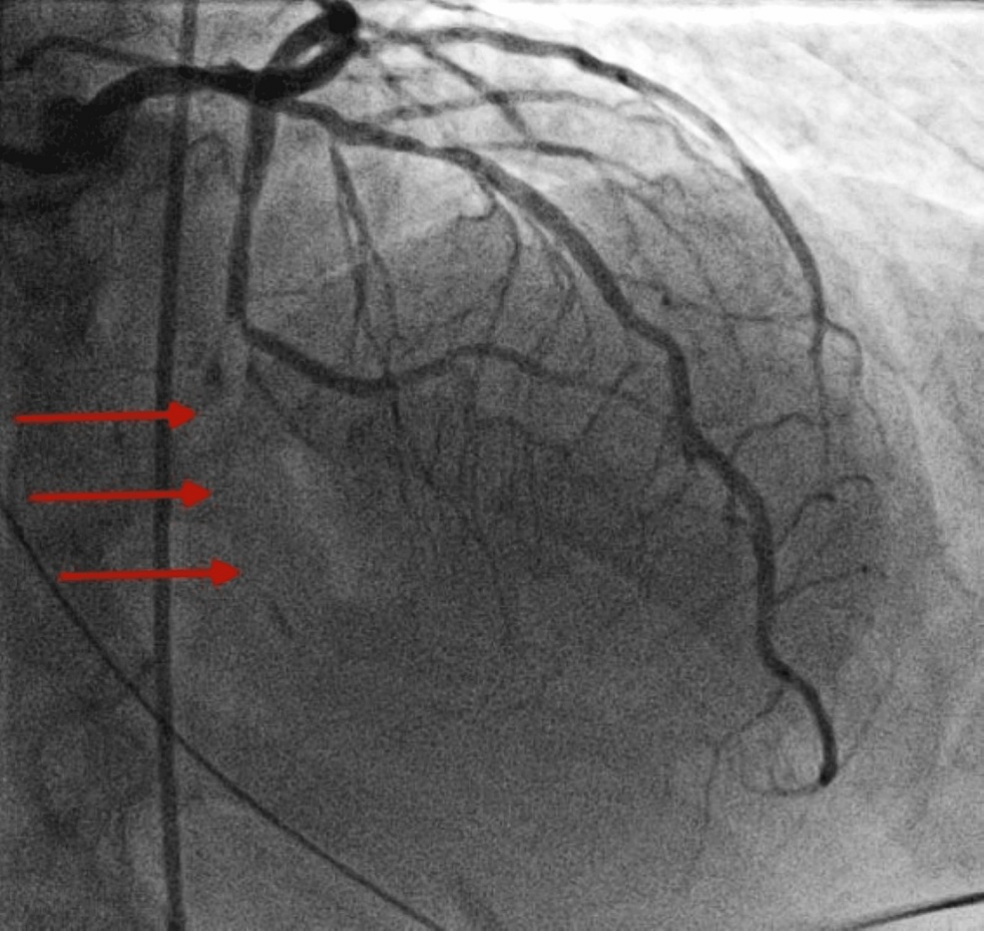
Left coronary angiography showing red arrows pointing at the occlusion site of the left posterior descending artery.

Cardiac catheterization indicated an occlusion in the distal segment of the left posterior descending coronary artery with the appearance suggesting the rupture of a coronary plaque (Figure 4).

The left ventricular ejection fraction remained preserved. Given the distal artery blockage impacting only a small proportion of the myocardium, PCI was not performed. Instead, the focus was shifted to treating the occluded middle cerebral arteries.

**Figure 5:**
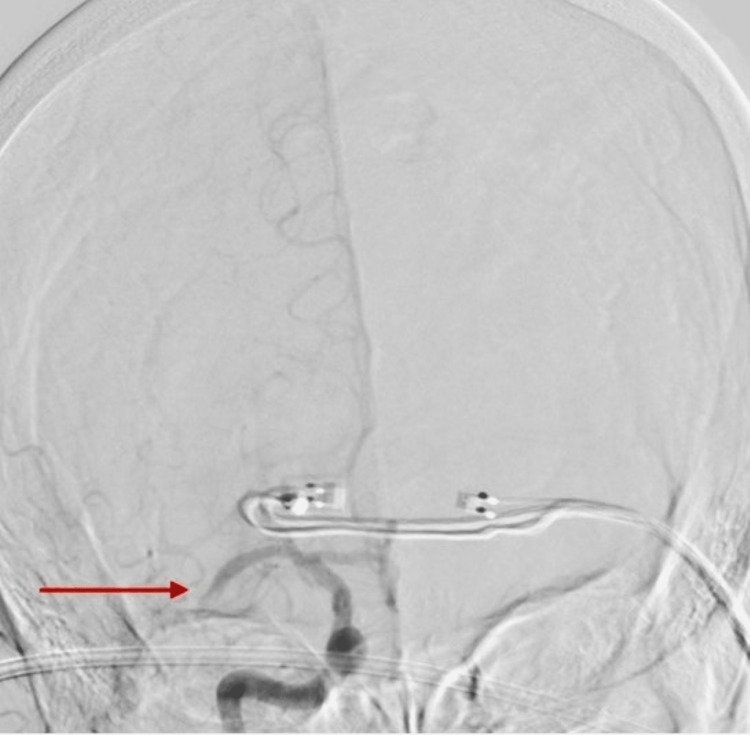
Right cerebral angiogram showing a red arrow pointing at the occlusion of the right middle cerebral artery.

**Figure 6:**
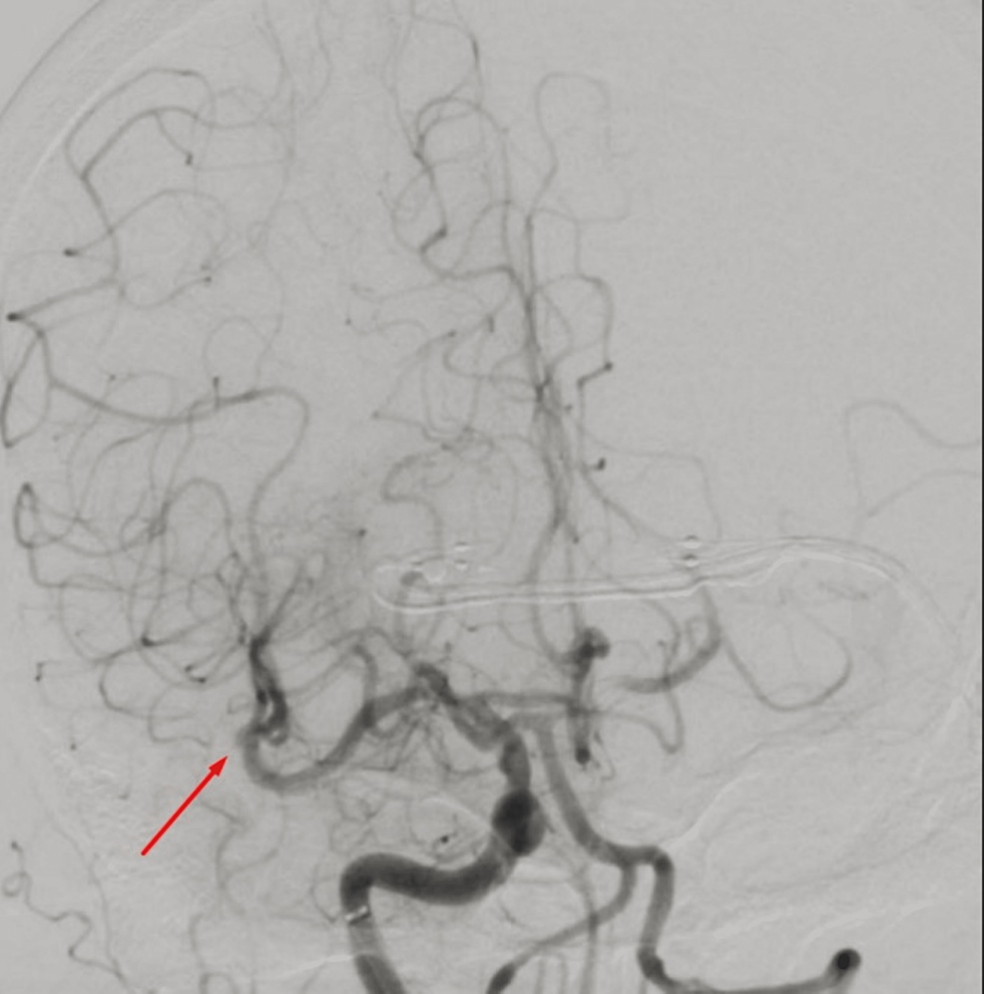
Right cerebral angiogram showing restoration of the blood flow in the right middle cerebral artery.

**Figure 7:**
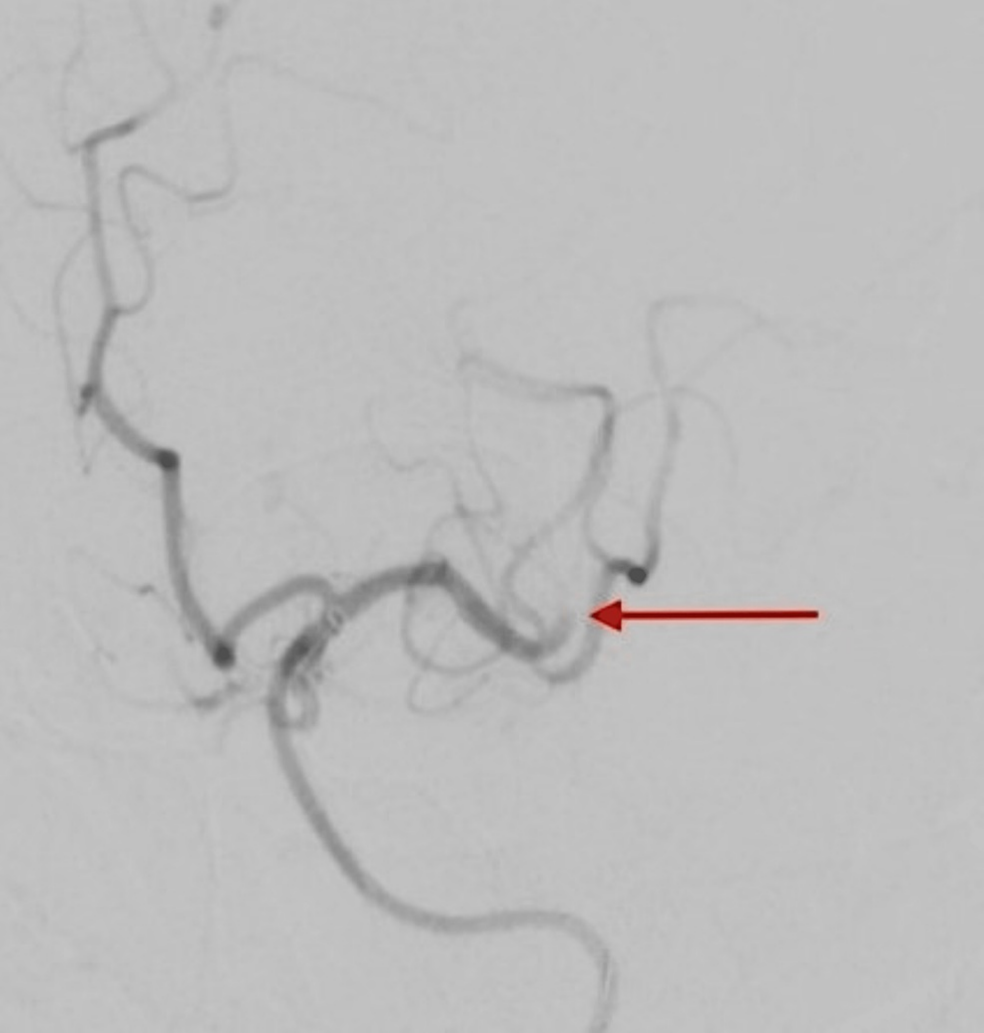
Left cerebral angiogram showing a red arrow pointing at the occlusion of the second branch of the left middle cerebral artery.

**Figure 8:**
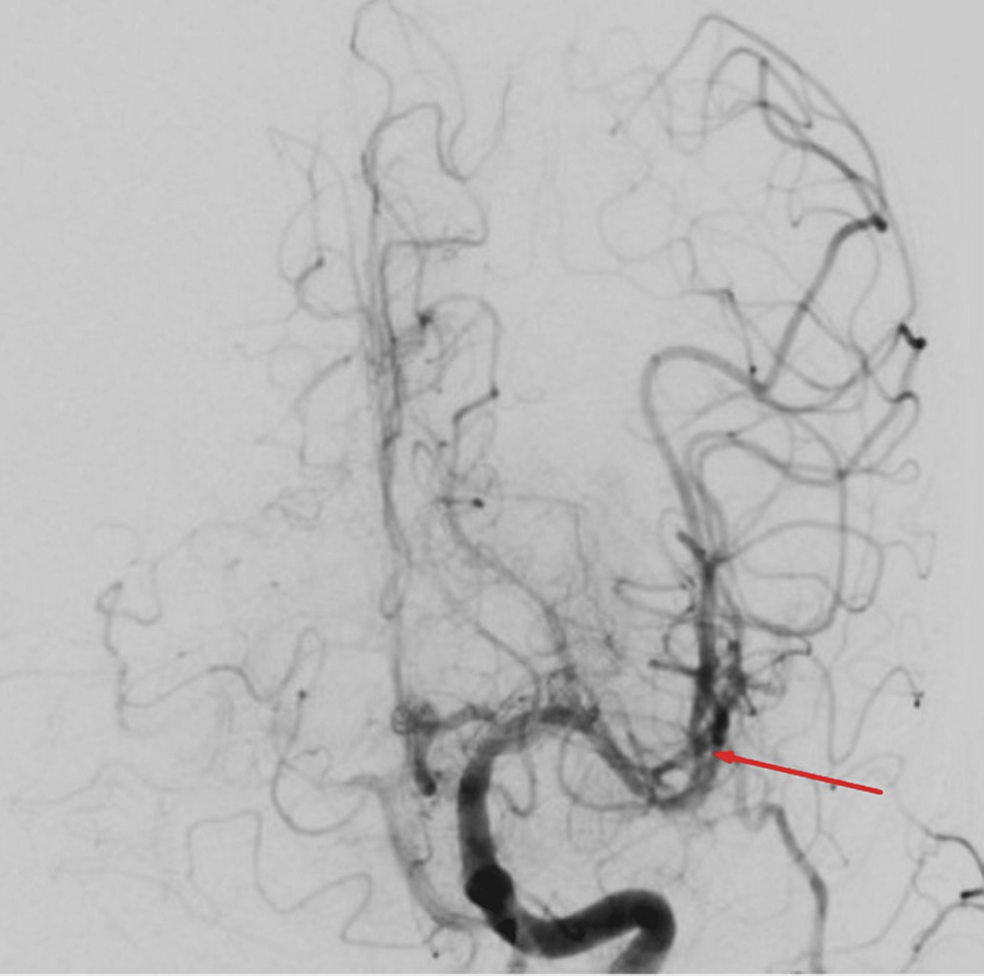
Left cerebral angiogram showing restored blood flow of the second branch of the left middle cerebral artery.

Occlusion of the right middle cerebral artery main branch underwent endovascular thrombectomy using a combination of Trevo Stentriever (Stryker, Kalamazoo, MI, USA) and aspiration, resulting in successful recanalization with final Thrombolysis in Cerebral Infarction (TICI) score of 2b (Figures 5-6), followed by endovascular thrombectomy using aspiration technique of left middle cerebral artery second branch with successful recanalization and final TICI score of 3 (Figures 7-8).

The patient was transferred to the medical intensive care unit (MICU) where she had an echocardiogram that showed a dilated and hypocontractile right ventricle with McConnell’s sign (Video 1). There was no evidence of shunt after agitated saline contrast injection.

**Video 1:** Transthoracic echocardiogram. An apical four-chamber view showing McConnell’s sign, characterized by hypokinesis of the mid-free wall of the right ventricle and hyperkinetic motion of the right ventricular apex (arrow).

**Figure 9:**
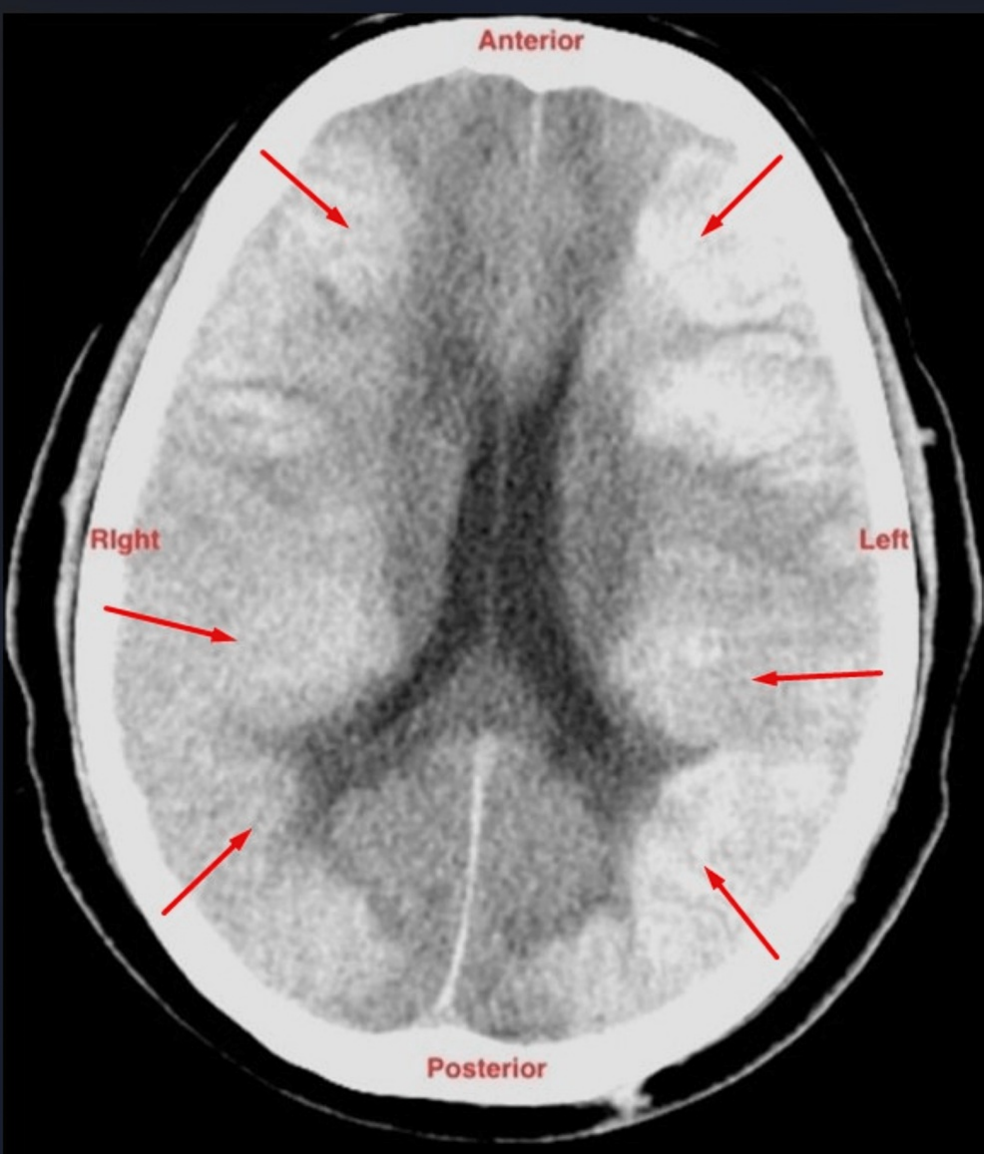
CT scan of the head revealing the presence of a bilateral subarachnoid hemorrhage. CT, computed tomography

Following the thrombectomy, a CT scan of the head was performed. The scan revealed the presence of a bilateral subarachnoid hemorrhage (Figure 9).

Anticoagulants and antiplatelets were held. In the ensuing hours, she lost brain reflexes. The caloric test, along with the apnea test, yielded positive results, indicating brain death. After careful consideration, the patient's family decided to discontinue life support.

## Discussion

This paper discusses the exceptional case of simultaneous STEMI, AIS, and acute pulmonary embolism. Such a convergence of conditions presents considerable diagnostic and therapeutic challenges for the medical team.

The understanding of the link between AIS and AMI was broadened following a study by Chin et al., which demonstrated that 12.7% of patients with AIS also experienced an associated AMI within 72 hours of hospital admission [3]. The treatment protocol for an infarction, where one vascular territory is affected before the other, primarily concentrates on addressing the initial event, followed by appropriate management of the subsequent event as it transpires. The term *cardio-cerebral infarction* (CCI), coined by Omar et al. in 2010, refers to the simultaneous occurrence of AIS and AMI [4]; however, the ideal strategy for the immediate management of a concurrent occurrence of both AIS and STEMI is not clearly outlined in evidence-based guidelines or clinical practice. The co-occurrence of acute pulmonary embolism along with AIS and STEMI adds a layer of complexity to both the comprehension and treatment of these conditions. While individual treatment protocols for each of these conditions are well-delineated in medical guidelines, their simultaneous presentation poses a significant therapeutic conundrum.

The manifestation of this triad has been reported in connection with systemic diseases like systemic lupus erythematosus, vasculitis, paraneoplastic syndrome, or COVID-19 infection. Alternatively, a paradoxical embolism, originating from a single source in both venous and arterial circulation, could also serve as a differential diagnosis for this uncommon triad of conditions [2,5]. The mechanism behind this perilous scenario is not fully comprehended and could potentially be influenced by multiple factors. For a systemic disease pathway, it could be secondary to endothelial dysfunction [6], hypercoagulability related to the type and treatment of cancer [7], and an antiphospholipid antibody syndrome [8]. In systemic diseases, a thrombus can form in both arterial and venous circulation. Therefore, while a paradoxical embolism could potentially occur, it is not a necessary condition for the simultaneous involvement of all three regions. When paradoxical embolism is present, and the source is on the arterial side, AMI, particularly infarction of the anterior and apical wall that is associated with reduced left ventricular systolic function, creates a foundation for the formation of a mural thrombus in the left ventricle [9]. These thrombi, which form post-AMI, are particularly susceptible to a heightened risk of embolization [10,11] and may explain simultaneous CCI and PE via the left-to-right trajectory of the embolus.

In patients with atrial fibrillation, embolization to both coronary and cerebral arteries has also been documented [12]. Similarly, a paradoxical embolus originating from a right ventricular thrombus or deep vein thrombosis through a patent foramen ovale is also a possibility [13,14]. The adrenergic surge associated with an AIS can lead to catecholamine-induced myocardial stunning. This is a frequent cause of stress-induced cardiomyopathy, also known as Takotsubo syndrome, which can mimic symptoms of STEMI, and, in turn, this scenario could promote the formation of an intracardiac thrombus, which has the potential to embolize the cerebral and coronary arteries [15,16] and pulmonary artery through left-to-right shunt.

In the case of our patient, it seems that several mechanisms were at play concurrently. Based on the patient's symptoms, it seems that AMI was the initial event that occurred. The form of the coronary occlusion observed during the coronary angiography points to an atherosclerotic cause, rather than it being the result of an embolic event. Venous thromboembolism leading to acute pulmonary embolism is likely related to a prolonged 13-hour flight. The World Health Organization states that the risk of venous thromboembolism doubles after engaging in long-distance air travel that lasts more than four hours [4,17]. Without evidence of a right-to-left shunt, the occurrence of coronary and cerebral infarction cannot be rationalized on the grounds of a paradoxical embolism. Undiagnosed and untreated atrial fibrillation was likely the source of the embolization that resulted in the occlusion of the bilateral middle cerebral arteries.

Irrespective of the underlying mechanisms, such a combination of acute conditions can be fatal due to the narrow therapeutic time window associated with each of them. Consequently, focusing on the management of one condition might result in a critical delay in treating the others. Implementing PCI before cerebral reperfusion therapy could elevate the risk of a substantial cerebral infarction that could lead to severe neurological deficits, cerebral edema, and potentially, death. The use of antiplatelet medications, given to our patients for initially planned PCI, is harmful for patients with large cerebral infarction and a substantial risk of hemorrhagic transformation [4,18]. 

Most of the existing research on managing such conditions consists of case reports or case series. For the cardio-cerebral infarction case series, a meta-analysis demonstrated that 10 out of the 44 enrolled patients died within a median of two days, despite aggressive management strategies. These included stenting in 15 patients, PCI without stenting in eight patients, coronary artery thrombectomy in eight patients, and endovascular thrombectomy for stroke in 10 patients [19].

As a result, despite the clinical demand, there are no evidence-based guidelines available for managing the co-occurrence of these conditions. The further accumulation and analysis of data about this clinical scenario could illuminate the best treatment strategies and help enhance outcomes for patients with such concurrent conditions.

## Conclusions

The concurrent manifestation of AMI, AIS, and acute pulmonary embolism is exceedingly rare. All three conditions are life-threatening, necessitating immediate medical intervention. The handling of such a multifaceted scenario is not well-researched. Despite significant clinical needs, there are no evidence-based guidelines available for managing the co-occurrence of these conditions. Additional research is required to enhance the outcomes for patients experiencing this rare convergence of conditions.
